# Whatever can go wrong, need not go wrong: Open Quality approach for epidemiology

**DOI:** 10.1186/s12982-021-00098-0

**Published:** 2021-07-17

**Authors:** Sandra Alba, Masja Straetemans

**Affiliations:** KIT Royal Tropical Institute, Amsterdam, The Netherlands

**Keywords:** Quality management, Quality assurance, Quality control, Juran, Donabedian, ISO 9000, Epidemiology

## Abstract

Quality assurance is one of the most important aspects of an epidemiological study, as its validity is largely determined by data quality. The mounting success of quality management in the industrial sector caused a rapid spread throughout manufacturing industries and beyond. Yet, little has been published so far on quality assurance in epidemiology. In this article we review three models for quality assurance (Juran, Donabedian and ISO 9000) and showcase how these can be brought together in one intuitive, systematic and flexible approach to quality assurance in epidemiology. The resulting Open Quality approach refers back to the three processes identified by Juran (planning, control and verification). During the planning stage, we propose a subdivision of the study process in a set of steps and a definition of quality attributes corresponding to activities in that step as suggested by the ISO approach. We refer to the Donabedian model to determine the level at which the control/monitoring should take place—structure, processes or outcomes. Along with an overview of the Open Quality approach we propose an Open Quality tool to support the definition of quality attributes, failure modes, preventive strategies, verification activities, and corrective actions, which form the backbone of the Open Quality approach.

**Supplementary Information:**

The online version contains supplementary material available at 10.1186/s12982-021-00098-0.

## Introduction

*Whatever can go wrong, will go wrong—*Murphy’s law supposedly explains why our data files get corrupted before being backed-up, and why the printer breaks down the day we planned to print all questionnaires for a survey. But beyond pessimistic predicaments, Murphy’s law also offers a very useful starting point for quality assurance. As we demonstrate in this article, systematically reflecting on “*what can go wrong?*” and crucially “*what can we do to prevent it?*” is the basis a very versatile approach to data quality assurance.

Quality assurance is one of the most important aspects of an epidemiological study, as the validity of study results is largely determined by data quality. High measurement error can dilute existing associations (Type II errors), while biased data can lead to incorrectly reporting associations (Type I errors) [1]. Quality management is a widely encompassing term and refers to management processes geared towards producing high quality results (e.g. products or services). While the foundations of quality management were laid during the industrial revolution, the discipline came of age in the 1950’s when Japan decided to make quality improvement a national imperative as part of rebuilding the country’s economy. A number of methods and tools have emerged since then, rooted in a variety of disciplines, including industrial engineering, information management and statistics. The mounting success of quality management in the industrial sector caused a rapid spread throughout manufacturing industries and beyond—to the service sector, government, and non-profit enterprises.

Yes, little has been published on quality assurance and quality control in epidemiology. Much of the literature on quality assurance in health sciences has arisen in the context of health care [2, 3] and clinical trials [4, 5]. In epidemiology most of the work related to quality assurance has been published in the realm of research integrity, in the form of guidelines for good epidemiological practice [6–10] and guidelines for reporting epidemiological studies [11]. A few epidemiological studies have published their own study-specific quality assurance and quality control procedures [12–14]. These descriptions represent a very rich source of activities and protocols that can be adopted or adapted in similar studies. However these reports typically do not embed their descriptions in the wider quality assurance frameworks, which can limit their applicability in more diverse settings.

In this article we present three purposefully selected models for quality assurance and discuss their relevance for epidemiological studies. The first is one of the earliest models proposed for quality assurance in industrial engineering (Juran model), the second is the most widely accepted quality assurance model in health care (Donabedian model), and the third is currently the most widespread quality accreditation model in the EU (ISO 9000). We reflect on these models’ basic principles and tools, and showcase how these can be brought together in one intuitive, systematic and flexible approach—and accompanying tool—to quality assurance in epidemiology.

## Overview of models and tools for quality assurance

### Juran model

The American engineer Joseph Juran (1904–2008) is one of the founders of quality management. Interestingly, he was more influential in Japan than in the United States at first. He visited Japan in 1950’s and consulted managers and engineers in managing for quality. Juran acknowledged that there are a number of definitions of quality, and that the meaning of quality also has a bearing on the approach to quality management. Juran’s approach to quality management first introduced in 1951 [15] comprises three managerial processes: quality planning, quality control, and quality improvement. These three processes have since come to be known as the Juran trilogy [16] and are described below.

*Quality planning* provides the process, methods, tools, and techniques to create a high quality product. There are 6 steps in this process: (1) Establish the project by providing the goals, direction, and materials, equipment, skills etc.; (2) identify all customers; (3) identify customer needs to inform product design; (4) develop product; (5) develop a process capable of delivering the product as it was designed, consistently, time after time; (6) develop process controls that keep the process operating at its full capability. Juran advocates the use of preventive risk analysis tools during the planning stage to inform process design (Box 1). *Quality control* provides stability. Quality control processes evaluate actual performance, compare actual performance to goals, and take action on the difference. After improvements have been made, a new level of performance has been achieved. *Quality improvement* aims to attain “unprecedented levels of performance”. At this point processes and goals are in place, though products may not all meet the goals. The approach therefore consists of “(1) discovering the causes—why do some products meet the goal and others do not—and (2) applying remedies to remove the causes”.

Juran has proposed a number of definitions for quality, two of which can be readily applied for epidemiological studies and data: ‘fitness for use’ and ‘freedom from deficiencies’ [16]. While freedom from deficiencies is a very noble goal to work towards, it may remain unattainable. The relative nature of ‘fitness for use’ on the other hand is very pragmatic and has gained traction in many fields, including data quality attributes [17] (see Box 2). The preventive nature of the Juran model lends itself well to applications in epidemiological studies. Quality control mechanisms are developed early on, during the planning stage of the study. They are closely linked to both quality goals and feedback loops. As such, the study becomes a dynamic learning system which facilitates prompt mid-course corrections. The strong focus on customers and customer needs feel artificial in research settings, although in theory research funders and society could be thought of as ‘the customers’. The Juran model has a strong focus on reducing waste and bringing systems to ever higher levels of performance. Interestingly this resonates well with the current scientific discourse around replicability, where “correctable weaknesses” in the design, conduct, and analysis of research studies are framed as a “waste” of valuable resources.

### Donabedian model

The physician Avedis Donabedian (1919–2000) is considered the authority in all matters concerning healthcare quality. In his seminal 1966 paper ‘Evaluating the Quality of Medical Care’ [3] he introduced a model to assess quality of care focussing on the ‘triad’ of structure, process and outcome. Structure refers to “the conditions under which care is provided” (facilities, equipment, human resources, organizational characteristics, etc.). Process are the “activities that constitute health care” (diagnosis, treatment, prevention, etc.). Outcomes are “changes in individuals and populations that can be attributed to health care” (changes in health status, knowledge, behaviour, satisfaction etc.).

In his later work he also describes a system for quality assurance [2], defined as “activity by which we obtain information about the level of quality produced by the health care system and, based on an interpretation of that information, take the actions needed to protect and improve quality”. Proposed steps are as follows: (1) Determining what to monitor (e.g. choice of tracers); (2) Determining priorities in monitoring; (3) Selecting an approach to assessing performance (structure, process and/or outcomes); (4) Formulating criteria and standards (see Box 2); (5) Obtaining the necessary information (surveys, records, observation); (6) Choosing when to monitor (prospective, concurrent or retrospective monitoring); (7) Choosing how to monitor; (8) Constructing a monitoring system; (9) Bringing about behavior change (re-adjustments and educational/motivational activities).

The Donabedian model has gained widespread acceptance in health care. Being health-related, it can seem like a natural choice of reference for epidemiologists. In many ways it can work very well, since the implementation of epidemiological studies can also be broken down into structures (human resources and equipment), processes (data collection, data management, data analyses) and outcomes (completed questionnaires, raw data, clean data, analyses outputs such as tables and graphs). A monitoring focus on structures would only make sense for studies of extended duration (e.g. surveillance systems or repeated surveys) with long term structural investments. For one-off studies, the focus may be more on processes and outcomes, as there is little scope for corrections ‘after the fact’. In these cases, concurrent monitoring activities are arguably more important.

### ISO 9000 models

The International Organization for Standardization (ISO) was founded in 1947 to reach world-wide uniformity in measurements. Thereafter it moved towards publishing standards. In 1987 ISO created the ISO 9000 family of standards for quality management, designed to help organisations ensure that they meet the needs of customers and other stakeholders while meeting statutory and regulatory requirements related to a product or service. The ISO 9000 standards have undergone a number of revisions since their creation, and over time have moved away from product inspection and towards a process-oriented approach in order to produce the sought after quality outcomes [18]. ISO 9000:1994 emphasized quality assurance via preventive actions, based on concepts of risk assessment and risk mitigation using tools such as the failure mode and effects analysis (Box 1). These concepts are very much in line with the Juran approach to quality management. The process oriented structure was introduced in ISO 9001:2000 edition, and was further developed in the ISO 9001:2015, as described below.

The process oriented approach of ISO 9001:2015 describes an organization as consisting of a series of interacting processes [18]. A process is a set of activities that uses resources (people, machines, etc.) to transform inputs into outputs. The output of one process is the input of another process, which stresses the importance of not treating each process in isolation (department, job, etc.). Each process needs to ensure it delivers (outputs) what the next process needs (inputs). The ISO 9001 Standard is designed to manage and improve processes with the following steps: (1) Identify your key processes; (2) define standards for those processes. (3) Decide how the process will be measured and evaluated; (4) document your approach to achieving the desired quality, as determined by your measurements; (5) continuously improve.

The ISO approach to quality assurance can be adapted to suit epidemiological research. However the focus on processes can feel unsatisfactory, since the data quality (per se an outcome rather than a process) should be the primary concern of quality assurance in epidemiology. One perceived advantage is that research organisations can get ISO certified by third-party certification bodies, which brings credibility to outside parties that standardised and documented processes are being followed. To qualify for ISO certification, an organisation must write a specific, step-by-step description for each process, and then demonstrate that it follows these procedures. However ISO certification has been criticised for being time-consuming and expensive (ISO standards are proprietary and certification relies heavily on external audits). Moreover, there is no evidence that ISO certification actually improves quality [19].

**Table:** Box 1. Risk analysis

Failure Mode and Effects Analysis (FMEA) or Hazard Analysis and Critical Control Points (HACCP) are two examples of risk analysis tools to identify potential weaknesses in a process. Procedures for conducting FMEA were described in US Armed Forces Military Procedures document MIL-P-1629 in 1949. During the 1970s, use of FMEA and related techniques spread to other industries. HACCP is the adaptation of the FMEA to the food industry
The aim of these analyses is to identify all possible failures or hazards in each part of a system, during its design stage, in order to ensure that they can be prevented from occurring in the first place. Applied to an epidemiological study, this can be done by systematically questioning, for each step in the survey process (e.g. study preparation, data collection, data analysis, etc.): what can go wrong (failure modes in FMEA, hazards in HACCP)? How can this be prevented? How can we check that we are doing things right (detection in FMEA)? How can we fix things if they go wrong (mitigation in FMEA and corrective actions in HACCP)?

**Table:** Box 2. Standards, criteria and attributes

Criteria and standards are “the tools by which the quality is measured” [2]. As such, they form the back-bone of many quality assurance approaches. However, there is no agreed-upon usage for these terms, and in fact, various contradictory definitions have been given [20]
Donabedian [2] defines a **criterion** as “an attribute of structure, process, or outcome that is used to draw an inference about quality. […] For example a criterion of outcome could be case fatality”. **Standards** are defined as “a specified quantitative measure of magnitude or frequency that specifies what is good or less so. […] For example a standard for case fatality could be: no more than 0.1% for a specified procedure (or a set of procedures) in a specified category of patients
**Quality attributes** according to Donabedian [2] are the “product characteristics [which] taken singly of in a variety of combinations constitute a definition of quality and, when measured in one way or another will signify its magnitude”. According to this definition data quality attributed in epidemiology refer to data quality framework dimensions such as relevance; accuracy; credibility; timeliness; accessibility; interpretability; and coherence [17]. These can either be attributes of the system that produced the data (i.e. the process) or of the data itself (data output/outcome) [21]
Donabedian also proposes a useful link between the standard-criteria duo and quality attributes: “Criteria and standards are vehicles by which quality attributes are translated to actual measurements”

Items in bold in this table and in the text can be traced back to this box as a reference

## The Open Quality approach for epidemiology

Based on the Juran, Donabedian and ISO models, we propose an approach for quality assurance specifically tailored to epidemiological studies. From of the three models reviewed, we selected elements that were most applicable to an epidemiological study and aimed for a user-friendly and intuitive approach. Our approach refers back to the three processes identified by Juran (planning, control and verification). This allows for a strong preventive focus, designed to prevent errors from arising in the first place. The first planning phase is therefore the most comprehensive phase. During the planning stage, we propose a subdivision of the study process in various steps and focusing on quality attributes affected by activities in each step, as suggested by the ISO approach. These activities can then be subject to a risk analyses inspired by the FMEA/HACCP tools. These analyses will inform the design of both survey methodologies as well as quality control (using Juran’s terminology) or monitoring activities (Donabedian terminology). The Donabedian model can be used to determine at which level the control/monitoring should take place—structure, processes or outcomes. The second and third Juran phases consist of control and improvement. Similar approaches have been described for these two phases by the Juran, Donabedian and ISO model: conducting control/monitoring activities and acting upon results.

**Fig. 1:**
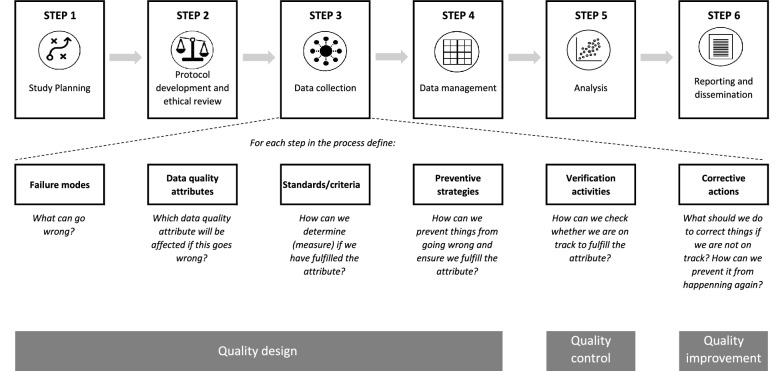
Schematic overview of the Open Quality tool

We provide an overview of the Open Quality approach in Table 1 and present a schematic overview of the Open Quality tool in Fig. 1. The Open Quality tool supports the definition of quality attributes, failure modes, preventive strategies, verification activities, and corrective actions, which form the backbone of the Open Quality approach. In Table 2 we show how this tool can be applied in a household survey, by focusing on the data collection step for illustrative purposes. We referred the OECD **data quality attributes** in for this example (accuracy, credibility, timeliness, accessibility, interpretability and coherence) [17]. An Excel template to apply the Open Quality tool all throughout a study is presented in Additional file 1.

**Table 1:** Overview of Open Quality approach

Phase 1: Design	1. Identify the steps in the study process, e.g.: (1) Study planning; (2) Protocol development and ethical review; (3) data collection; (4) data management; (5) data analysis; (6) Reporting and dissemination [10] 2. Identify **failure modes** in each step of study process and affected **data quality attributes** by referring to a chosen framework for data quality (e.g. OECD’s relevance, accuracy, credibility, timeliness, accessibility, interpretability, and coherence [17]) 3. Define **standards or criteria** [2] for each data quality attribute as a measurable assessment of whether the data quality attribute is fulfilled 4. Identify **preventive strategies** and **verification activities** to address each failure mode*.* Verification activities can assess study structure (equipment and personnel), processes (adherence to plans) and outcomes (data) 5. Develop survey methodology, including manuals (training manual, field manual, standard operating procedures) and plans (data management plan and statistical analysis plan) in line with the preventive strategies and verification activities
Phase 2: Control	6. Conduct **verification activities** and document outcomes
Phase 3: Improvement	7. Promptly address failures modes with **corrective actions** so that they do not compromise the overall study quality (mid-course corrections, re-adjustments and educational/motivational activities) 8. Implement changes in methodology to prevent failure modes from occurring in the future

Items in bold in this table and in the text can be traced back to this table as a reference

**Table 2:** Application of the Open Quality tool in an a household survey (data collection step)

Failure modes	Data quality attributes	Standards/criteria	Preventive strategies	Verification activities	Corrective actions
*1. What can go wrong?*	*2. Which data quality attribute will be affected if this goes wrong?*^a^	*3. How can we determine (measure) if we have fulfilled the attribute?*	*4. How can we prevent things from going wrong and ensure we fulfill the attribute?*	*5. How can we check whether we are on track to fulfill the attribute?*^b^	*6. What should we do to correct things if we are not on track? How can we prevent it from happening again?*^c^
Data fabrication	Accuracy (and credibility)	All fields in the questionnaire should be filled in with information genuinely observed or provided by the respondents	Use tool for electronic data collection (EDC) with tablet, program start and end time of the interview and collect GPS location of households visited to be reviewed regularly by survey team	100% daily review of questionnaires by supervisors Random spot-checks by supervisor 10% household revisited/call back survey independently by a team of independent monitors	Replacing teams that are not functioning well (with 'reserve' interviewers not part of the initial team)
Interviewers do not fill in questionnaire completely	Completeness	Only completed questionnaires shall be uploaded	Built-in EDC functionality whereby data cannot be sent if questionnaire is incomplete Built in EDC functionality whereby cannot proceed to next questions if all previous not completed		
Interviewers do not visit all households in the sample (only those that are easier to access)	Completeness	All sampled households should be visited except if they are in a cluster excluded from the sample for security reasons	Daily plans for each supervisor, submitted to field managers	100% review of incoming data on weekly basis compared to targets	Immediate contact with survey manager in case targets are not being met
Responses to various questions are not coherent	Coherence	Answers to related questions should be coherent [select related questions]	Built in EDC functionality with consistency checks between responses to selected questions, prompting interviewer to double-check responses and ask for clarifications to the respondent		
Data cannot be uploaded to the server (no internet connection)	Accessibility (and timeliness)	Data should be uploaded on the same day or next day at the latest	Each tablet has a sim-card with data bundle to send data in case wireless connection cannot be obtained	100% review of incoming data on weekly basis compared to targets	Immediate contact with survey manager in case targets are not being met

^a^Here we refer to the OECD data quality dimensions [17] but other data quality frameworks can be used

^b^This field may not always be applicable

^b^Only applicable if a control practice is defined

While Table 2 has been compiled for the data collection step only for illustrative purposes, the same logic can be followed for all other steps in the implementation of a study—or survey in our example. It is important to realise that the relative importance of the different data quality attributes will vary depending on the process steps. For example during planning, relevance and timeliness are important attributes—“*what can go wrong*” is that the planned survey does not provide the information needed for data users within the timeframes for decision-making. Useful **preventive strategies** include *c*onsulting with data users during study preparation to ensure that all the study meets their data needs and discussing the timelines with various stakeholders. Interpretability is key when developing a questionnaire (e.g. in the protocol development and ethical review step) to ensure that interviewers and/or interviewees understand the questions in the same way as the investigators had intended when developing them. Pilot and field testing tools and discussions with pilot interviewers and interviewees thereafter are good strategies to ensure a good alignment between everybody’s understanding of questions. Accuracy, completeness and coherence are paramount during data management, which is where data management plans including strategies such as double data entry, consistency checks, skip patterns and unique identifiers come in. Similarly to the data collection example in Table 2, spot checks on a database sample—e.g. against original paper forms—can be a useful **verification strategy***.* Accuracy is again key during data analysis. Statistical analyses plans can help prevent certain mistakes, but multiple analyses by independent analysts—for either the entirety or a selection of study outputs—may be needed as additional verification. Finally, when preparing to disseminate a study, interpretability and accessibility of outputs will be the main consideration, which is why user-specific dissemination products may be needed, and may need to be developed in consultation with representatives of given user groups.

There are three important practical considerations when filling in the Open Quality tool. First, the tool is best filled in and developed in consultation with all staff (or their representatives) involved in the study, and possibly external stakeholders (e.g. funders or other external collaborators). Indeed, the process of filling in the tool and associated discussions to reach consensus amongst all parties can help ensure trust and ownership of the quality assurance process by all and ensure that the strategies are acceptable and feasible in practice. Second, various iterations between **failure modes** and **quality attributes** may be needed to define both satisfactorily, and one failure mode may relate to a combination of quality attributes (e.g. credibility and accuracy or accessibility and timeliness in Table 2). The conversation about issues in a certain process step may start with the question “*what went wrong here last time?*” (failure mode) or “*how do we ensure complete/accurate/coherent data?*” (quality attribute), before moving on (or back) to filling all other entries in the tool (standards/criteria, preventive strategies, verification activities, corrective actions). Third and last, it is important to bear in mind that **corrective actions** follow from **verification activities**, meaning that there are no corrective actions if there are no verification activities. Furthermore, both verification activities and corrective actions may not always be necessary or possible (as can be seen in Table 2 those last two columns were not always filled in). Data collection, data management and data analysis are process steps which typically need several verification activities and corrective actions.

## Conclusion

The Open Quality approach is an intuitive, systematic and flexible approach to data quality assurance—*intuitive* because it builds upon investigators’ knowledge of what can go wrong (often based on experience has gone wrong in the past); *systematic* because it entails a step-by-step reflection and documentation of all potential quality threats and mitigation strategies in given study; *flexible* because it provides investigators the freedom to tailor their approach to their needs and the possibility not to act upon all threats (so long as this is a calculated and transparent decision).

We have chosen the term ‘Open Quality’ for the framework and tool presented here as we believe that it fits in the ‘Open Science’ vision to ensure transparent and accessible research. Open science is heralded as one answer to the replicability crisis, by ensuring total transparency in the research process and making sure that all material is available to replicate study results. Open Science encompasses at least four concepts: open access (online free peer-reviewed research articles); open data (online publication of research data); open source (use of software which anyone can use and change from existing source code); and open methodology (sharing explicit and detailed procedures before and after the study has been conducted) [22]. The Open Quality framework and tools offers both a systematic approach for quality assurance for *data producer*s, but also clear documentation for *data users*. Open disclosure of the chosen approach for quality assurance using tools such as the one presented here is foundational to both open data and open methodology as it provides all the necessary information for users to assess the data and methodology’s fitness for use—where users includes those who will be appraising study results, those who will be taking public health or personal decisions based on the study data, as well as those who will re-use the data for secondary analyses.

*Whatever can go wrong, need not go wrong.* On a bad day it can be tempting to see Murphy’s law as governing the universe and perversely mocking us towards failure. But exactly in such days, it is important to remember that the famous saying attributed to Murphy—an American engineer working for the United States Air Force in the 1950s—was most likely meant as a reminder to fellow team members to be cautious and make sure everything was accounted for. The universe may seem to be working against us at times, but a lot of optimism can be taken from learning from past mistakes and—at least sometimes—preventing things from going wrong.

## Supplementary Information

**Additional file 1.** Open quality tool template.

## Data Availability

Not applicable.
